# Supplementary material for: Another Look at Obesity Paradox in Acute Ischemic Stroke: Association Rule Mining
Source: J Pers Med. 2021 Dec 29;12(1):16. doi: 10.3390/jpm12010016 (PMC8781183; doi:10.3390/jpm12010016)
Supplement: Supplementary file 1 [file jpm-12-00016-s001.zip › jpm-1448712-supplementary-FC.pdf]

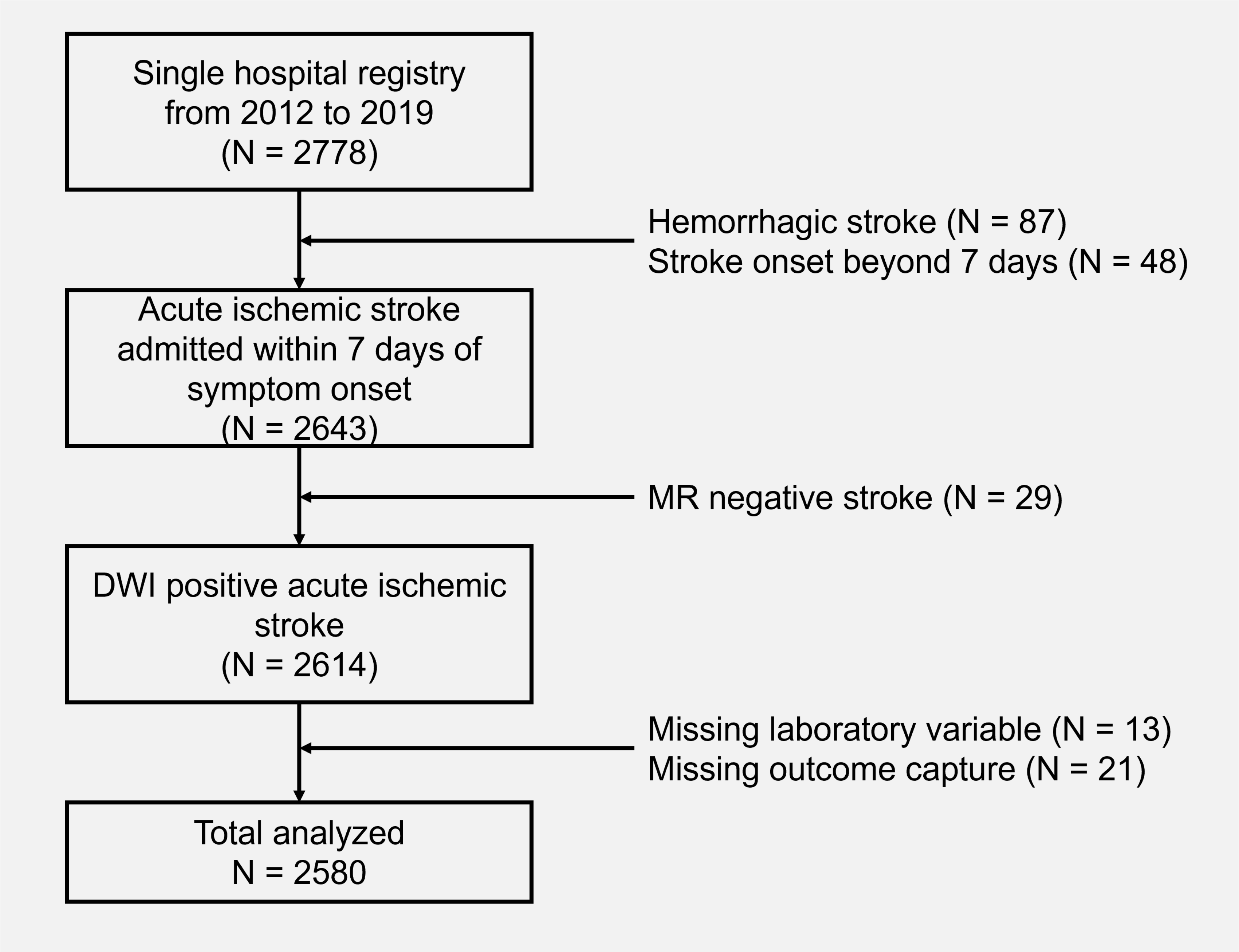

Figure S1. Flow chart of the participants. MR, magnetic resonance; DWI, diffusion-weighted image.

Table S1. Results of binary logistic regression analysis for predictors of poor functional outcome at 3 months in patients with acute ischemic stroke, considering the variable age and BMI as continuous values.

	Univariate OR (95% CI)	P	Multivariate OR (95% CI)	P
Age, years	1.06 (1.05-1.07)	<0.001	1.96 (1.74-2.21)	<0.001
BMI, kg/m^2	0.92 (0.90-0.94)	<0.001	0.92 (0.83-1.01)	0.090
Stroke Severity, NIHSS				
Mild (0-5)	0.14 (0.11-0.18)	<0.001	0.11 (0.08-0.15)	<0.001
Moderate (6-14)	1.00 (reference)	-	1.00 (reference)	-
Severe (>14)	2.44 (1.60-3.72)	<0.001	2.45 (1.56-3.86)	<0.001
Diabetes	1.49 (1.25-1.77)	<0.001	1.69 (1.37-2.10)	<0.001
Current Smoking	0.48 (0.40-0.58)	<0.001	0.71 (0.55-0.91)	0.006
**Thrombolysis**	**1.94 (1.51-2.49)**	**<0.001**	**0.69 (0.49-0.98)**	**0.039**
**Cardioembolism**	**1.74 (1.44-2.11)**	**<0.001**	**0.71 (0.55-0.92)**	**0.009**
Hypertension	1.67 (1.41-1.98)	<0.001	1.05 (0.85-1.31)	0.650
Hyperlipidemia	0.97 (0.79-1.21)	0.811	0.91 (0.70-1.18)	0.466
Men	0.57 (0.48-0.67)	<0.001	0.98 (0.79-1.22)	0.870

OR, odds ratio; CI, confidence interval; BMI, body mass index; NIHSS, National Institute of Health Stroke Scale.

Table S2. Results of binary logistic regression analysis for predictors, considering the interaction between the stratified age and BMI, of poor functional outcome at 3 months in patients with acute ischemic stroke.

		Multivariate OR (95% CI)	P
Age, years			
18-54		0.77 (0.41-1.44)	0.4062
55-64		1.00 (reference)	-
≥65		2.78 (1.76-4.39)	<0.001
BMI, kg/m²			
Underweight (<18.5)		1.14 (0.27-4.69)	0.8602
Normal weight (18.5-22.9)		1.00 (reference)	-
Overweight (23.0-24.9)		0.93 (0.52-1.68)	0.8212
Obese (≥25)		1.18 (0.68-2.05)	0.5614
Stroke Severity, NIHSS			
Mild (0-5)		0.11 (0.08-0.15)	<0.001
Moderate (6-14)		1.00 (reference)	-
Severe (>14)		2.33 (1.49-3.64)	<0.001
Diabetes		1.62 (1.31-2.01)	<0.001
Current Smoking		0.68 (0.53-0.87)	0.0021
Thrombolysis		0.65 (0.46-0.91)	0.0138
Cardioembolism		0.75 (0.58-0.96)	0.0244
Hypertension		1.14 (0.92-1.41)	0.2349
Hyperlipidemia		0.90 (0.70-1.17)	0.4248
Men		0.93 (0.75-1.15)	0.4793
BMI, kg/m²	Age, years	Interaction OR	Interaction P
Underweight (<18.5)	18-54	0.99 (0.13-7.45)	0.9926
Underweight (<18.5)	≥65	1.63 (0.36-7.36)	0.5268
Overweight (23.0-24.9)	18-54	1.23 (0.49-3.08)	0.6558
Overweight (23.0-24.9)	≥65	0.81 (0.42-1.56)	0.5325
Obese (≥25)	18-54	0.51 (0.21-1.24)	0.1371
Obese (≥25)	≥65	0.63 (0.53-0.87)	0.1402

OR, odds ratio; CI, confidence interval; BMI, body mass index; NIHSS, National Institute of Health Stroke Scale.

Table S3. Results of the frequent pattern growth algorithms of four different dataset (BMI: Obese, Overweight, Normal, Underweight) with independent and dependent variables in patients having acute ischemic stroke.

		LHS															RHS			
		Age (τ)			Severity		Hypertension		Gender	Mechanism	Hyperlipidemia	Thrombolysis	Smoking		Diabetes					
	Rule	$\tau < 55$	$55 \leq \tau < 65$	$65 \leq \tau$	Mild	Severe	Yes	No	Male	Non-CE	No	No	Yes	No	Yes	No		Support	Confidence	Lift
Obese	1	O			O							O					Good	0.2000+	0.9617	1.3628
(N = 880)	2	O			O												Good	0.2068++++	0.9578	1.3574
	3	O										O					Good	0.2022++	0.9417	1.3345
	4				O						O		O				Good	0.2056+++	0.9234	1.3086
	5	O															Good	0.2136+++++	0.9126	1.2932
Overweight	1		O		O					O		O					Good	0.1610++++	0.8943	1.3192
(N = 683)	2				O				O	O	O	O	O				Good	0.1420+	0.8818	1.3008
	3				O				O		O	O	O				Good	0.1522+++	0.8813	1.3001
	4		O		O							O					Good	0.1771+++++	0.8768	1.2934
	5				O			O	O	O		O				O	Good	0.1449++	0.8761	1.2923
Normal	1				O			O	O			O				O	Good	0.1423+	0.8707	1.4496
(N = 899)	2				O			O	O		O	O					Good	0.1601+++	0.8622	1.4355
	3				O			O	O			O					Good	0.1724++++	0.8611	1.4335
	4				O			O	O							O	Good	0.1501++	0.8598	1.4315
	5				O			O	O								Good	0.1824+++++	0.8497	1.4146
Underweight	1				O								O				Good	0.0847+	0.8333	2.0921
(N = 118)	2			O		O											Poor	0.1610++++	1.0000	1.6619
	3					O	O										Poor	0.1101+++	1.0000	1.6619
	4					O											Poor	0.1864+++++	0.9565	1.5897
	5			O	O									O	O		Poor	0.0932++	0.8461	1.4062

LHS: left hand side; RHS: right hand side; BMI: body mass index; CE: cardioembolism

O: represents the group indicated.

+: indicates the degree of the support for the rule
